# Diagnosis and Conservative Management of Ureteral Orifice Injury During Robotic Prostatectomy for a Large Prostate with a Prominent Median Lobe

**DOI:** 10.1089/cren.2018.0109

**Published:** 2019-05-30

**Authors:** Fevzi Bedir, Murat Keske, Saban Oguz Demirdogen, Huseyin Kocaturk, Ali Fuat Atmaca, Abdullah Erdem Canda

**Affiliations:** ^1^ Department of Urology, University of Health Sciences, affiliated with the Erzurum Training and Research Hospital, Erzurum, Turkey.; ^2^ Department of Urology, University of Health Sciences, affiliated with the Kayseri Training and Research Hospital, Kayseri, Turkey.; ^3^ Department of Urology, Ankara Yildirim Beyazit University, School of Medicine, affiliated with Ankara Ataturk Training and Research Hospital, Ankara, Turkey.; ^4^ Department of Urology, Koc University, School of Medicine, Istanbul, Turkey.

**Keywords:** prostate cancer, robotic prostatectomy, ureter orifice injury, median lobe

## Abstract

***Introduction:*** Robot-assisted laparoscopic radical prostatectomy (RALRP) is now considered the standard treatment for localized prostate cancer. However, challenges may arise when dealing with large prostates with a prominent median lobe because the ureteral orifices may not always be visible during dissection and maybe injured in the process. We describe our experience on the diagnosis and conservative management of ureteral orifice injury in this situation.

***Case:*** A Gleason score 3 + 3 prostatic adenocarcinoma was detected during 12-quadrant prostate biopsy performed after measurement of a serum prostate specific antigen value of 8.1 ng/mL in a 65-year-old man presenting with lower urinary tract symptoms. The left ureter orifice was observed to have been injured by scissors at dissection of the neck of the bladder and enlarged median lobe at RALRP. An online video call was made to more experienced robotic surgeons for advice. Diagnosis and management of the ureteral injury are presented.

***Conclusion:*** Ureteral orifice injury during an RALRP may be managed conservatively with intraoperative ureteral stenting without the need for reimplantation nor conversion to open surgical techniques. Online video call with experienced robotic surgeons is helpful in the decision process.

## Introduction

Robot-assisted laparoscopic radical prostatectomy (RALRP) has become a minimally invasive technique widely used in the curative treatment of localized prostate cancer. Owing to its technical advantages and low complication rates, the robotic system has been used in ∼80% of prostatectomy procedures in the United States in the past 10 years.

Ureter injuries during RALRP are rare, with a reported incidence between 0.1% and 0.3%.^1^ This complication may result in additional postoperative morbidity, prolonged hospital stay, and secondary treatment requirements.

The purpose of this report was to describe intraoperative diagnosis and treatment of left ureter orifice injury occurring during bladder neck dissection in a patient with enlarged prostate undergoing RALRP.

## Case

**FIG. 1.**
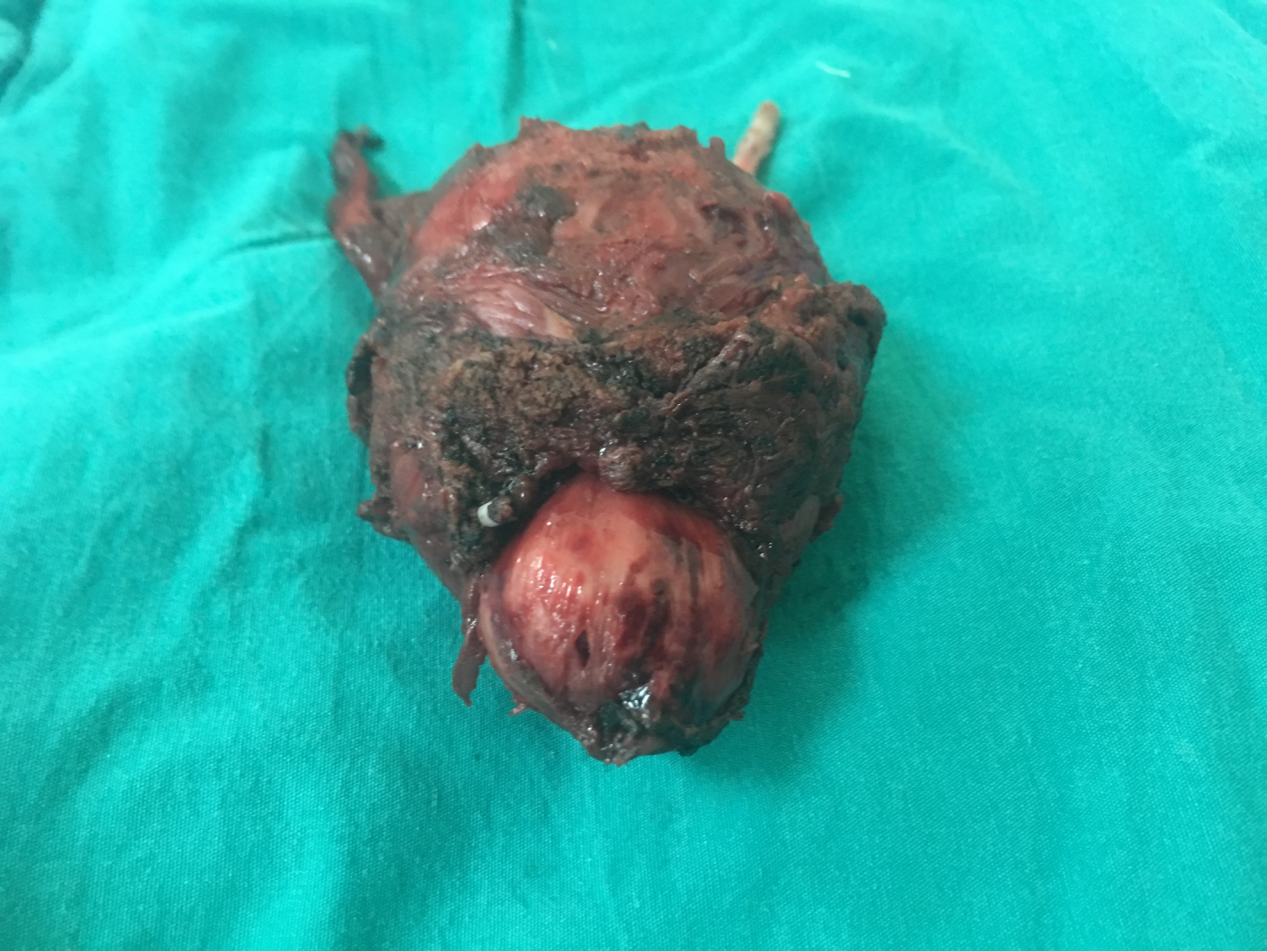
Radical prostatectomy specimen.

**FIG. 2.**
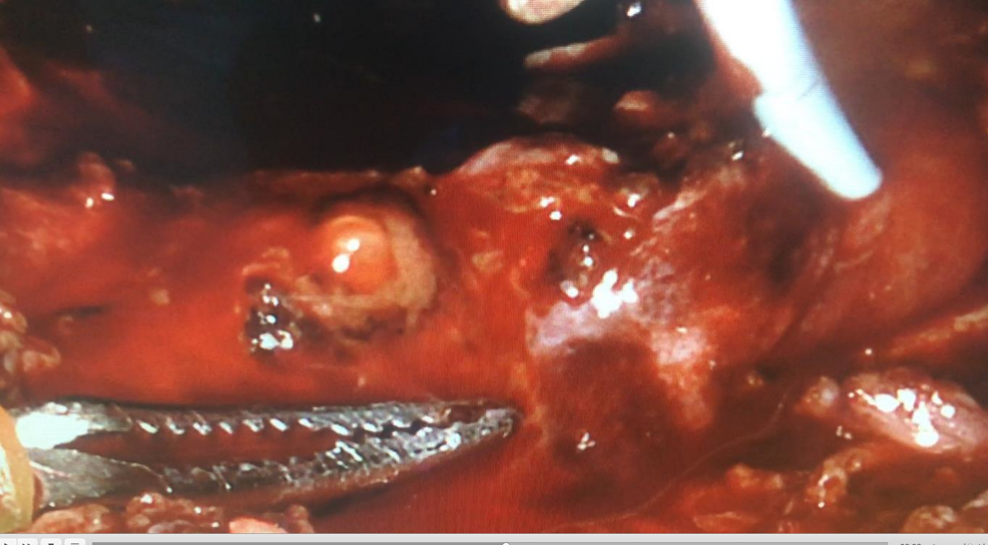
Urinary drainage from the injured left ureter orifice.

**FIG. 3.**
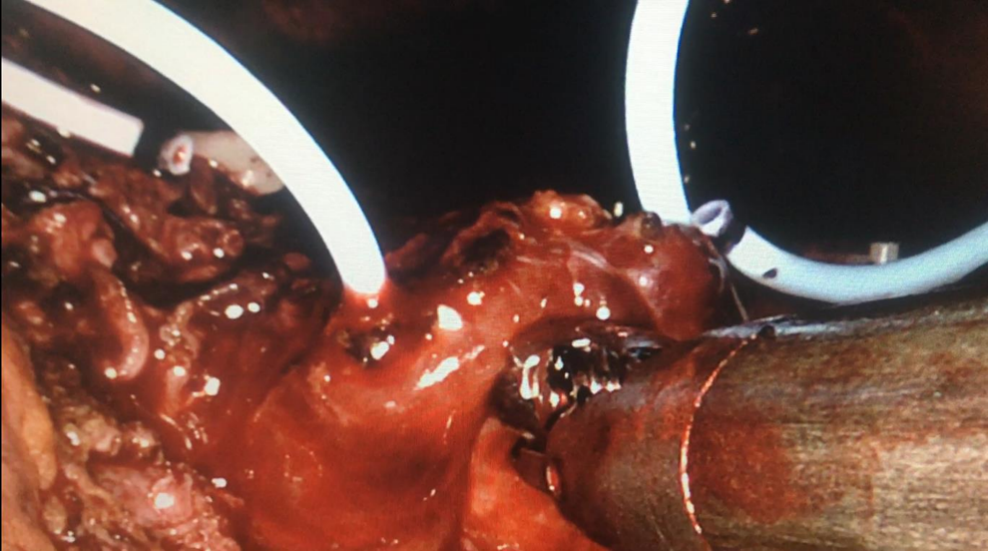
Double-J stent implantation to both ureter orifices.

A Gleason score of 3 + 3 prostatic adenocarcinoma was detected at 12-quadrant transrectal ultrasound (TRUS)-guided prostate biopsy performed after determination of a serum prostate specific antigen value of 8.1 ng/mL in a 65-year-old man presenting with lower urinary tract symptoms. Prostate volume was calculated at 202 g at TRUS. No lymph node of pathologic size was determined at multiparametric prostate MRI. During RALRP, a 2/0 Vicryl suture was applied on the median lobe for traction to supply a better view for bladder neck and exposure of the large median lobe. We subsequently observed an injury of the left ureter orifice occurred during dissection with monopolar scissors. At inspection, the right ureter orifice was natural in appearance, whereas the left orifice had lost its natural appearance in association with a laceration. However, peristalsis and urinary flow from the area thought to be the left ureter orifice were observed. Online video call was made with experienced colleagues who were in another city (A.F.A. and A.E.C.) and the surgical fields on the screen were presented to them to get advice. Owing to their suggestion, two 4.7F 28 cm Double-J stents were inserted through the assistant port into the abdomen and installed in the both ureteral orifices, and RALRP was completed. The second orifice was also stented to prevent injury (Figs. 1–3). Postoperative prostate weight was measured as 202 g. Estimated blood loss was 250 mL. During control ultrasonography (USG) performed on postoperative day 1, the bilateral kidneys were normal, and no hydronephrosis was observed. Postoperative follow-up was uneventful and the patient was discharged on day 7. Cystography was performed on day 21 that showed no leakage and urethral catheter was removed. Control USG was performed periodically during hospitalization and no hydronephrosis was observed. The pathology report was pT2a with negative surgical margins. The Double-J stents were removed with the assistance of a flexible endoscope on the second month. The ureter orifices remained behind the neck of the bladder with normal appearance.

## Discussion

Ureter injuries are rare complications during RALRP. Reported risk factors for ureter injuries include a history of infection, previous transurethral resection of the prostate, previous abdominal surgery, previous transurethral resection of the prostate, radiotherapy, extended pelvic lymphadenectomy for high-risk patients, and enlarged prostate and median lobes.^2^

Various therapeutic methods can be applied, depending on the level and length of ureter injury. Jhaveri et al. reported performing robotic ureteroneocystostomy on one of three patients with ureter injury, open transuretouretostomy on the second, and treated the third using primary anastomosis.^3^ Krasnow and coworkers adopted a conservative approach in a case of ureter injury developing after RALRP, and treated this with percutaneous drainage and antegrade ureter stent insertion.^4^

We report a case of ureter orifice injury diagnosed intraoperatively and treated with retrograde Double-J stent inserted through an assistant port. We think that the presence of a large median lobe prevented observation of the ureter orifice during dissection, and that this led to the occurrence of injury. To prevent injury, a better image might have been obtained at this stage with a 30° down scope, rather than a 0° lens that we used. Owing to the absence of tactile sensation in robotic surgery, it is very important for the operation to be performed with good observation. The amount of bleeding in our case, with its high prostate volume, was 250 mL. This was higher than the mean estimated value in our series. To have a better vision, we applied a Vicryl suture on the median lobe and used it for upward traction. We think that proximity of the median lobe to the ureteral orifices and the bleeding that occurred during dissection also played a role in the injury. Two aspirators can be used if necessary to observe the orifices more clearly for this purpose, particularly in the presence of an enlarged median lobe and bleeding.

Recording images of the operation is useful in terms of assessing complications by observing them again. Complications can be treated by sending these images to more experienced individuals during surgery and requesting their opinions and advice. This can be regarded as another advantage of endoscopic surgery.

In addition, we think that because of the proximity between the ureter orifices and the anastomosis line in cases of enlarged prostate, fixing the transurethral catheter using inflation constitutes a mechanical obstacle to urinary drainage. Our patient's transurethral catheter did not provide sufficient urinary drainage in the early postoperative period, but drainage reached adequate levels when the transurethral catheter balloon was deflated. Under such circumstances, and as in this case, we think that sufficient urinary drainage can be established and anastomosis leakage could be prevented by securing the transurethral catheter around the penis without its balloon inflated.

In conclusion, ureter orifices could be injured during RALRP particularly in patients with an enlarged median lobe. After diagnosis, this can be effectively treated with intraoperative Double-J stent implantation into the ureter. Online video call with experienced surgeons is easy, safe, and supportive.

## Abbreviations Used

MRI: magnetic resonance imaging
RALRP: Robot-assisted laparoscopic radical prostatectomy
TRUS: transrectal ultrasound
USG: ultrasonography

## References

[1] koc e, Canda AE Robotic urologic surgery complications. MIS 2018;2:7

[2] Teber D, Gözen AS, Cresswell J, Canda AE, Yencilek F, Rassweiler J Prevention and management of ureteral injuries occurring during laparoscopic radical prostatectomy: The Heilbronn experience and a review of the literature. World J Urol 2009;27:613–618 19513722 10.1007/s00345-009-0428-7

[3] Jhaveri JK, Penna FJ, Diaz-Insua M, Jeong W, Menon M, Peabody JO Ureteral injuries sustained during robot-assisted radical prostatectomy. J Endourol 2014;28:318–324 24147874 10.1089/end.2013.0564

[4] Krasnow RE, Wingo MS, Leveille RJ Identification and conservative management of a distal ureteral injury occurring during robotic-assisted laparoscopic radical prostatectomy. J Robot Surg 2008;2:261–263 27637798 10.1007/s11701-008-0109-3

